# Identifying novel transcription factors involved in the inflammatory response by using binding site motif scanning in genomic regions defined by histone acetylation

**DOI:** 10.1371/journal.pone.0184850

**Published:** 2017-09-18

**Authors:** Peter S. Askovich, Stephen A. Ramsey, Alan H. Diercks, Kathleen A. Kennedy, Theo A. Knijnenburg, Alan Aderem

**Affiliations:** 1 Center for Infectious Disease Research, Seattle, Washington, United States of America; 2 Department of Biomedical Sciences, Oregon State University, Corvallis, Oregon, United States of America; 3 Institute for Systems Biology, Seattle, Washington, United States of America; Chinese University of Hong Kong, HONG KONG

## Abstract

The innate immune response to pathogenic challenge is a complex, multi-staged process involving thousands of genes. While numerous transcription factors that act as master regulators of this response have been identified, the temporal complexity of gene expression changes in response to pathogen-associated molecular pattern receptor stimulation strongly suggest that additional layers of regulation remain to be uncovered. The evolved pathogen response program in mammalian innate immune cells is understood to reflect a compromise between the probability of clearing the infection and the extent of tissue damage and inflammatory sequelae it causes. Because of that, a key challenge to delineating the regulators that control the temporal inflammatory response is that an innate immune regulator that may confer a selective advantage in the wild may be dispensable in the lab setting. In order to better understand the complete transcriptional response of primary macrophages to the bacterial endotoxin lipopolysaccharide (LPS), we designed a method that integrates temporally resolved gene expression and chromatin-accessibility measurements from mouse macrophages. By correlating changes in transcription factor binding site motif enrichment scores, calculated within regions of accessible chromatin, with the average temporal expression profile of a gene cluster, we screened for transcriptional factors that regulate the cluster. We have validated our predictions of LPS-stimulated transcriptional regulators using ChIP-seq data for three transcription factors with experimentally confirmed functions in innate immunity. In addition, we predict a role in the macrophage LPS response for several novel transcription factors that have not previously been implicated in immune responses. This method is applicable to any experimental situation where temporal gene expression and chromatin-accessibility data are available.

## Introduction

Macrophages are long-lived coordinating cells of the innate immune system. Activation of tissue macrophages by Toll-like receptor (TLR) stimulation initiates a dynamic program of gene expression changes involving hundreds of genes that are associated with processes such as phagocytosis, antigen presentation, immunoregulation, and non-oxidative metabolism [1–4]. This gene expression program involves scores of transcription factors (TFs) whose activation is regulated both hierarchically [5–7] and temporally [7–9] and whose accessible binding sites in the genome change over time due to stimulation-dependent alterations in epigenetic state of the chromatin [7, 10, 11]. One of the key chromatin marks directing the transcriptional response to endotoxin stimulation in macrophages is histone acetylation (HAc), which is associated with open chromatin and active promoters [10, 12]. Functional TF binding sites (TFBS) are often found within regions of histone acetylation, and our previous work has shown that the binding sites within histone-acetylated regions tend to appear as distinct features in the quantitative signal that represents the local amount of HAc ChIP-seq fragment recovery [13].

Various systems biology approaches have been used to map the transcription factors that regulate the transcriptional response of macrophages and dendritic cells to stimulation with bacterial endotoxin lipopolysaccharide (LPS) [14, 15] including (i) promoter scanning of genes clustered by temporal expression profiles [1, 16, 17] to identify known TFBS position-weight sequence patterns (motifs) that are enriched within the gene cluster; (ii) time-lagged correlation analysis of TF gene expression and target gene expression [9] (which can detect TFs that are dynamically regulated at the transcript level, but not those that are exclusively post-translationally regulated); (iii) siRNA inhibition of selected TFs, with qPCR gene expression profiling of selected target genes [18]; (iv) p300-guided sequence analysis [11]; (v) high-throughput multiplexed ChIP-seq [7]; and (vi) expression quantitative trait locus (eQTL) profiling [19–21]. Motif-based scanning for enriched TFBS within gene promoters has yielded multiple insights into the TFs regulating macrophage activation [1, 9, 16, 22–24] but is unable to comprehensively define the relevant TFs because the analysis is generally constrained to the promoter-proximal sequence in order to yield a tractable number of candidate molecules [9, 25, 26]. However, mammalian TF binding sites are often distal to the transcription start site (TSS), for example, in enhancers that can be many kilobase distant [11, 13, 27, 28].

As more large biological data sets are deposited in public repositories, big data analytics is an increasingly useful tool for predicting TF binding sites, tissue distribution, function and interactions. This approach is promising and offers a number of advantages, such as the ability to comprehensively analyze large numbers of cells and tissues simultaneously, and to make specific predictions based on the complete picture. These predictions can then be sorted by probabilities and tested in the lab. Such a computational approach has been used to successfully predict key TFs that play a role in cell differentiation; for example, ectopic expression of just nine of the top candidate TFs for epithelial retinal pigment cells, was sufficient to transform human fibroblasts into retinal pigment epithelial-like cells [29]. Bioinformatic analysis of gene expression profiles, based on whole transcriptome sequencing data from 33 mouse tissues, was used to produce an online database of fundamental functional annotation for mouse TFs [30]. Such computational efforts are not limited the TF predictions. Recent work has demonstrated the ability to predict large numbers of lincRNAs from RNA Seq data [31].

In this work, we build on our previous finding that active TFBS are concentrated in local “valleys” of HAc that occur within HAc-enriched regions [13] and our previous analytical approach in which HAc valley signals at a single time point were used to inform TFBS enrichment analysis [24, 28]. Here we have analyzed temporal HAc measurements in LPS-stimulated primary macrophages in order to obtain TFBS motif-specific *temporal binding propensity profiles* that we correlated with temporal gene expression profiles. In contrast to single-time-point epigenome-guided analysis [24, 28] and TF-expression-to-target-expression correlation analysis [9], this approach enables the detection of TFs that regulate target gene expression without the assumption that TF expression reflects binding.

## Materials and methods

### Macrophage tissue culture and RNA isolation

All animal studies were approved by Center for Infectious Disease Research Institutional Animal Care and Use Committee. Murine bone marrow-derived macrophages (BMDMs) were cultured from female C57BL/6J mice (age 8–12 weeks) as previously described [9] and on day six, cells were re-plated into six-well tissue culture plates. On day seven, cells were incubated for the indicated times (see text in Results section) in complete RPMI with rhM-CSF and 10 ng/mL of LPS (from *Salmonella enterica* serovar minnesota R595; List Biological Laboratories, Campbell, CA) and then harvested. RNA was isolated using TRIzol (Thermo Fisher Scientific, Waltham, MA) following the manufacturer’s instructions.

### Microarray assay

For each sample, 1 μg of RNA was amplified and labeled using the Affymetrix single-step protocol and hybridized to Affymetrix Mouse Exon 1.0 ST Array GeneChips (Affymetrix, Santa Clara, CA). The GeneChips were scanned using the Affymetrix GeneChip Scanner 3000 and processed into probe-level intensity (“.CEL'') files using the Affymetrix GeneChip Operating Software. Array data files are available in GEO (GSE100059).

### Microarray data processing

Affymetrix exon array files were processed using the Affymetrix Power Tools software using probe-to-probeset mappings from the University of Michigan Custom CDF project (ENTREZG, release 18.0.0) (#%affymetrix-algorithm-param-apt-command-line = apt-probeset-summarize -a rma-sketch—pgf-file MoEx10stv1_Mm_ENTREZG_18.0.0.pgf—clf-file MoEx-1_0-st-v1.r2.clf—use-disk false -o outENTREZv18.0-LPS—cel-files celFilesLPS-BMDM.txt). Processed data were loaded into Analyst (GeneData, Basel, Switzerland). Expression levels for genes that have an intensity of at least 64 (log_2_ of 6) were analyzed by ANOVA. Permutation *q* values were determined using balanced permutations and the cutoff of permutation *q* value of 0.01 and fold change of 5 or greater was used to select significantly changing genes. Upregulated and downregulated genes were separated and clustered separately (Positive Correlation Distance 1–r).

### Histone-acetylation ChIP-seq data processing

BMDMs were prepared as described above and stimulated on day 7 with 10 ng/mL of LPS for the indicated times. Immunoprecipitation (IP) was carried out as described in [24] using a rabbit polyclonal IgG for the acetyl-H4 IP (Merck Millipore, Billerica MA; catalog number 06–866). A sequencing library for the Illumina Genome Analyzer was derived from the IP using the Illumina reagent kit as previously described in [32] and sequenced on the Genome Analyzer II (Illumina, San Diego, CA) with 36-cycle chemistry. Raw files from this study are available in GEO (GSE54414). Reads were aligned to the reference mouse genome (GRCm38) using GSNAP [33], sorted and indexed using samtools [34], converted to UCSC BED format using bedtools [35] bamToBed, deduplicated, 3’-extended by 122 bp using bedtools slopBed, converted to UCSC bedgraph format using bedtools genomeCoverageBed, and converted to Affymetrix BAR file format [36] using a custom script in the MATLAB computing environment version R2015a (Mathworks, Natick, MA).

### Valley score calculation

Valley scores were computed based on the HAc ChIP-Seq signal sampled at a resolution of 10 bp. First, the HAc ChIP-Seq signal was smoothed by convolving it with a Gaussian kernel with a standard deviation of 40 bp. Next, local minima of the HAc ChIP-Seq signal were identified: for each sample point, the maximum signal values in the windows 50–500 bp to the right and left of the point were computed using a sliding window approach. If the signal value at the sample point was less than 70% of the minimum of its two surrounding local maxima (to the right and to the left), this sample point was designated a “valley''. The “valley score'' assigned to this point is the minimum of these two local maxima. For all sample points that were not identified as a valley, the valley score signal was set to zero, thus reducing the data track to only the local minima of the HAc ChIP-Seq signal. The valley score calculation was implemented in MATLAB.

### TF ChIP-seq data processing

For the IRF1, IRF8, and SPI1 ChIP-seq datasets, we obtained SRA files from NCBI GEO (Accession Number GSE56121). SRA files were converted into FASTQ files, and filtered for quality and common adapter sequences. Filtered FASTQ files were aligned to the GRCm38 genome assembly (UCSC gene annotation build mm10) using GSNAP. We then used Subread featureCounts [37] to count reads within genomic features.

### ChIP-seq analysis

In order to test the ability of our algorithm to predict TFs regulating each temporal expression cluster, we compared the ChIP-seq counts for IRF1 or IRF8 in the promoter regions of genes in each temporal expression cluster to the average counts for that TF in 1000 randomly generated gene sets of equal size. For each target we computed the average and standard deviation of the log_2_-transformed counts within the range of -2000 to +500 bp with respect to the transcription start site across the 1000 random gene sets ("background average and standard deviation"). We then computed the log_2_ counts in the promoters of genes in the biologically-derived clusters and converted these into *z*-scores using the background average and standard deviation values for that ChIP-seq experiment type and cluster type. From the *z-*scores, we obtained *p* values using the area under both tails of the normal distribution above |*z*| and below -|*z*|. We then adjusted the *p* values for multiple hypothesis testing using the Benjamini-Hochberg false discovery rate method [38]. This analysis was implemented in the R statistical computing environment ([39, 40]; version 3.2.1).

### Time-lagged correlation analysis

For each combination of a transcription factor binding site (TFBS) motif and a gene expression cluster, we computed the Pearson correlation coefficient between two sets of samples: (i) the time-course motif Clover raw scores for the DNA sequence in AcH4-valley regions within ±5 kbp of the transcription start sites of the genes in the cluster; and (ii) the time-course, cluster-median expression data at time points corresponding to the time-points for the AcH4 experiments (0, 1, 2, and 4 h) plus a time lag *τ* (the time-lagged correlation *R*_*τ*_*)*. One fixed time lag *τ* was selected for each cluster by maximizing the sum-squared *R*_*τ*_ values for all motifs that were associated with the cluster by a Clover enrichment analysis at least one time point (allowing the time lag to take any value in the range 0–2 h). The gene expression data at arbitrary time points were obtained by linear interpolation of the cluster-median gene expression measurements at the sampling time points 0, 2, 4, and 12 h. The optimal time lags for each of the clusters are (in hours): DC1 2.0; DC2 1.72; DC3 2.0; UC1 2.0; UC2 1.44; UC3 2.0; UC4 2.0; UC5 0.976. This analysis was implemented in the R statistical computing environment.

### Gene regulatory network analysis

We used the Ingenuity Pathways Analysis (IPA) tool (QIAGEN Bioinformatics, Redwood City, CA) to query for experimentally validated TF-gene interactions.

## Results

### Gene expression dynamics in LPS-stimulated macrophages

**Fig 1:**
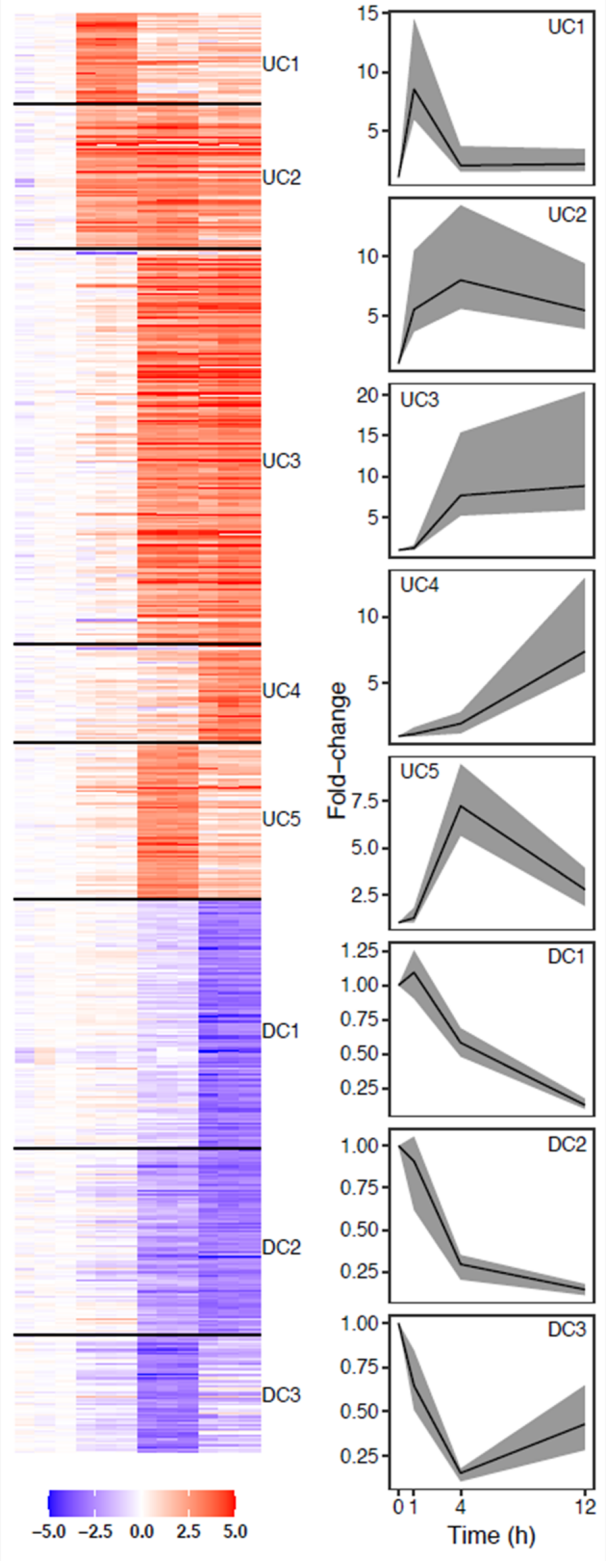
Transcriptional response to LPS. Heatmap of five upregulated and three downregulated clusters (left) and median cluster fold change (right). Lines show median cluster expression and shaded areas show interquartile range.

In order to identify transcription factors that regulate the macrophage response to stimulation with LPS, we first profiled the transcriptomes of mouse bone-marrow-derived macrophages (BMDMs) without stimulation and at 1, 4, or 12 hours post-stimulation using exon-targeted microarrays. Restricting the analysis to the most strongly LPS-responsive transcripts, we identified 707 that were differentially expressed at one or more time points (*q* value ≤ 0.01 and fold-change ≥ 5). Each differentially expressed gene was assigned to one of eight temporal expression profile clusters using a partitioning algorithm (*k*-means). Five clusters contain genes that were up-regulated in response to LPS, labeled Upregulated Cluster 1 (UC1) through Upregulated Cluster 5 (UC5), and three contain genes that were down-regulated, labeled Downregulated Cluster 1 (DC1) through Downregulated Cluster 3 (DC3) (Fig 1). Under the hypothesis that the distinct temporal patterns of gene expression are regulated by distinct sets of TFs [1, 9, 10], on a cluster by cluster basis, we analyzed DNA sequence in the 5' regulatory regions to identify TFBS for cluster-level enrichment analysis.

### Over-represented transcription factor binding motifs in gene promoter regions

**Fig 2:**
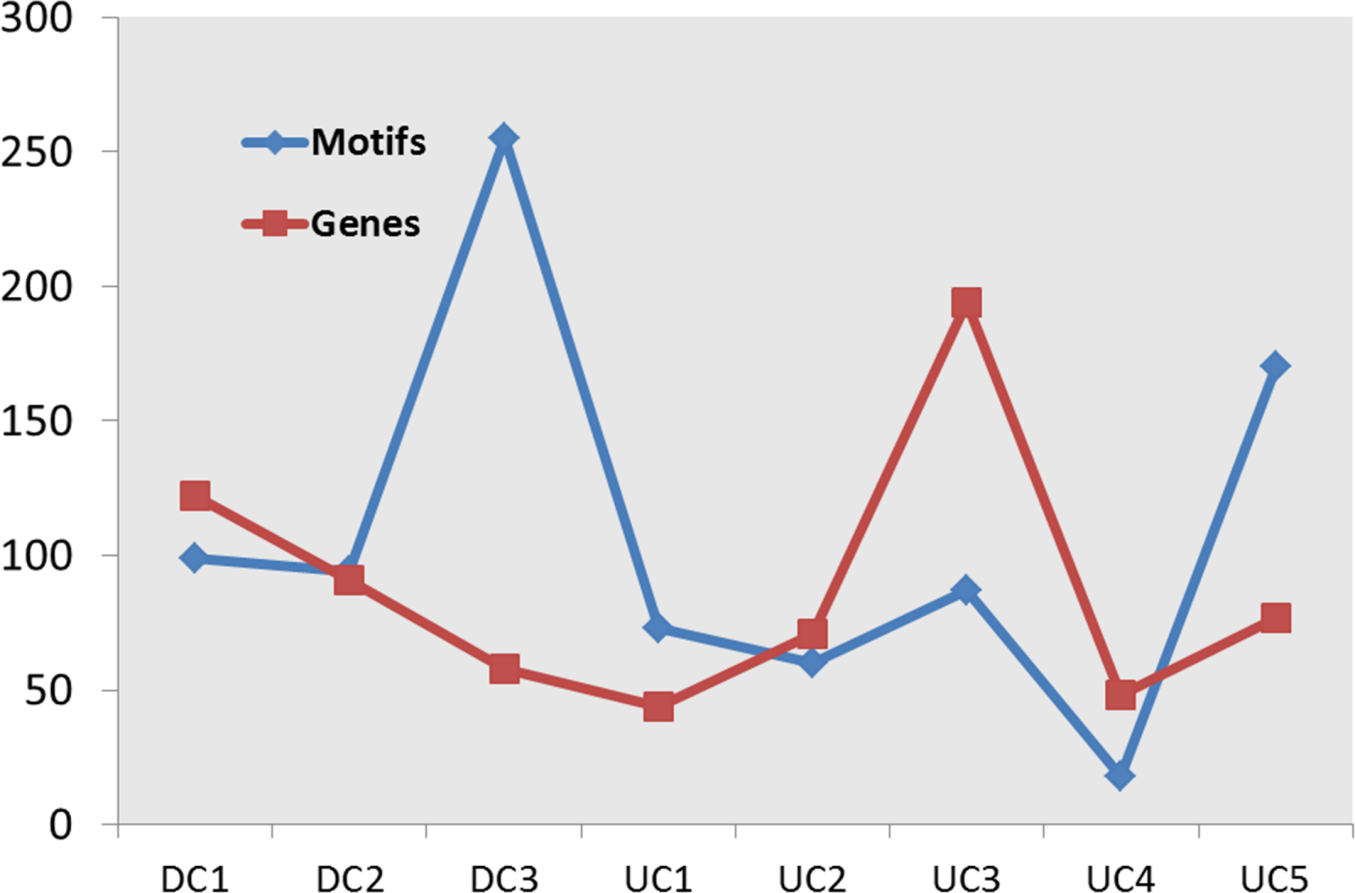
Promoter analysis of genes in eight clusters. Blue line shows the number of over-represented motifs (*p ≤* 0.01) in each cluster. Red line shows the number of genes in each cluster.

In order to identify cluster-specific TFs whose binding sites are enriched within the promoters of genes in each cluster, we computationally analyzed DNA sequence from 1,500 bp upstream to 500 bp downstream of the transcription start site of each gene. We then used the Clover tool [41] to score the enrichment of matches to TFBS motifs (from the TRANSFAC database) within the promoter sequences for the genes in each cluster as described previously [42] using the promoter regions of all macrophage-expressed genes as a background set. Across the eight clusters, the number of significantly overrepresented TFBS motifs (*p* ≤ 0.01) varied from 18 to 255 and did not correlate with the number of genes in the cluster (Fig 2). A high proportion of the TFs whose binding site motif matches were enriched are known to have a role in inflammation; for instance, the top 20 enriched motifs (by *p-*value) in cluster UC3 (Fig 1) include those for IRF, ISRE, TCF3, STAT6 and BLIMP1. This analysis also identified a significant number of TFs with no known role in the macrophage LPS response. For example, TFBS motif matches for TAL11, MYF and MZF1 were enriched in the promoter regions of genes in DC3.

A limitation of this approach is that the Clover scores and enrichment *p*-values for each cluster only suggest which TFs could be playing a role in regulating expression of that set of genes. Using these data alone, it is difficult to determine the relative contribution of each TF to the regulation of each cluster, and whether the TF is acting as an activator or as a repressor. In order to refine our predictions, we used temporal epigenetic profiling to further refine predictions for functional regulatory elements [43] in the activated macrophages.

### Defining active regulatory elements

Previous work from our group and others has established that the density of regulatory elements mediating macrophage responses to TLR stimulation is strongly enhanced within promoter regions marked by histone acetylation (HAc) [13, 24]. Specifically, the density of TF binding sites is increased within ~100 bp dips (valleys) in the HAc signal in noncoding genomic regions that are otherwise strongly histone-acetylated [13]. Incorporating HAc valley information into a motif-based TF binding site prediction algorithm, significantly improves accuracy [13]. Here, we have extended that approach to identify TFs that are associated with specific temporal programs of the macrophage transcriptional response to LPS stimulation.

**Fig 3:**
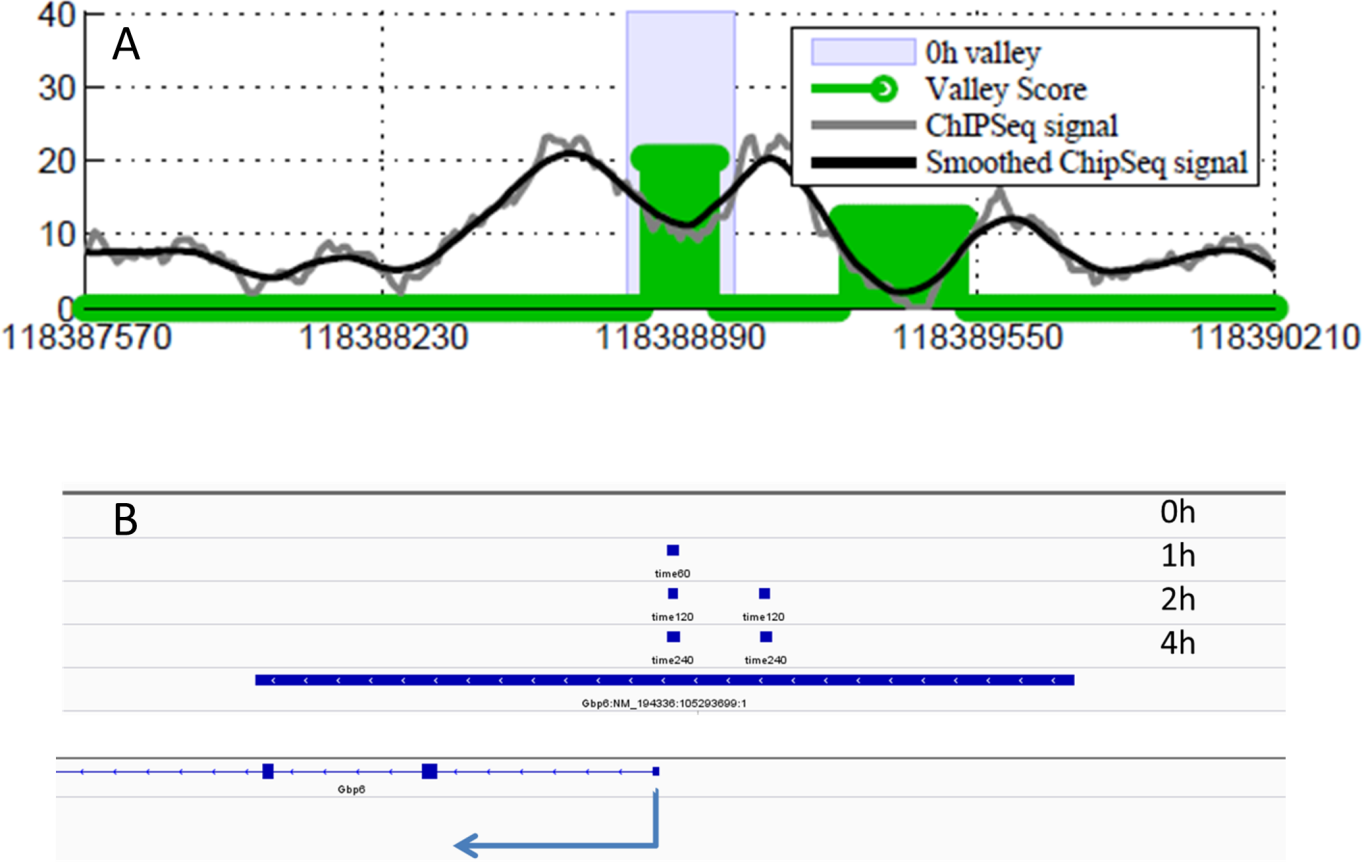
AcH4 valleys and active promoter regions. (A) AcH4 valleys. ChIP-seq signal and smoothed ChIP-seq signal are shown by gray and black lines respectively. Green bars represent the locations of detected AcH4 valleys. (B) Active promoter regions, defined as regions where detected AcH4 valleys (short blue bars shown for different time point) overlap with the ±5,000bp region around TSS (long blue bar).

Bone marrow-derived macrophages were harvested just prior to and at 1, 2 and 4 hours following LPS stimulation and histone-acetylated regions were mapped genome-wide by chromatin immunoprecipitation with high-throughput sequencing tag analysis (ChIP-seq) using an antibody against acetylated histone 4 (AcH4) [44] (using normal IgG as a control). For each time point, we computationally mapped AcH4-enriched regions genome-wide by comparing the local tag count in the AcH4 IP-based sample to the control sample and determined locations of the AcH4 valleys (Fig 3A); a total of 15,730, 12,623, 11,995, and 13,460 valleys were detected genome-wide in the 0, 1, 2 and 4 hour samples respectively. We defined the active promoter regions (APRs) as those AcH4 valleys within ±5,000 bp of the TSS of each gene (Fig 3B). Restricting APRs to this distance from the TSS is a tradeoff in order to effect a compromise between maximizing the number of candidate regulatory regions and unambiguous assignment of APRs to specific genes.

### Motif search and results

**Fig 4:**
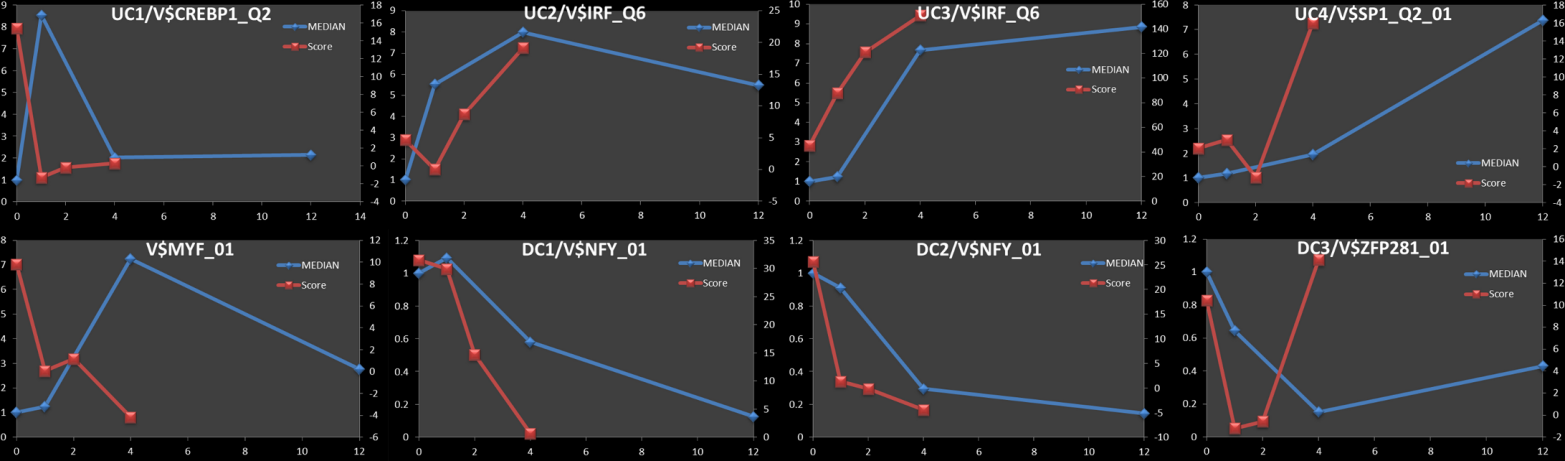
Top ranked motif for each of the eight expression clusters. Median fold change for each of the eight clusters is represented using blue lines and the values are shown on the left Y axes. Red lines represent Clover raw scores for the top ranked motif and the values are shown on the Y axes on the right. Based on a time-lagged correlation analysis (using optimum lag time for each motif), the correlation between the Clover score and the cluster-median expression levels are: UC1/V$CREBP1_Q2 - *R* = 0.828 (t = 2.0); UC2/V$IRF_Q6—*R* = 0.777 (t = 1.0004); UC3/V$IRF_Q6—*R* = 0.999 (t = 1.343); UC4/V$SP1_Q2_01—*R* = 0.8965 (t = 2.0); UC5/V$MYF_01—*R* = -0.900 (t = 1.001) DC1/V$NFY_01—*R* = 0.992 (t = 0.589); DC2/V$NFY_01—*R* = 0.916 (t = 2.0); DC3/V$ZFP281_01—*R* = 0.304 (t = 2.0).

We tested the sequence in APRs of each cluster at each time-point for over-representation of matches for each of 909 vertebrate TFBS motifs in the TRANSFAC database relative to a background of promoter sequences for all genes expressed in the entire dataset using a log-average-likelihood score (Clover [41]) as described previously [42]. Motifs with enrichment *p* ≤ 0.01 for at least one time-point were retained for further analysis. We interpreted the Clover raw score for each motif as a signal representing the strength of association of the corresponding TFs with the genes in the cluster and we hypothesized that temporal changes in this score indicated the time-dependent regulatory activity of the TF for the cluster. That is, we would expect that the changes in raw score for motifs corresponding to TFs regulating a significant number of genes in a cluster would correlate (either positively or negatively) with the temporal cluster-median expression profile. Therefore, we ranked the list of enriched motifs for each of the eight clusters by the magnitude of the change in score across all time points (max*(score)–*min*(score)*) (S1 Table). Fig 4 shows the median fold-change (blue lines) and the Clover raw score (red lines) for the top ranked motif in each cluster. Although the temporal resolution of these data does not allow for the precise timing of specific features of expression level dynamics for each cluster (e.g., maximum point), it would be expected that the binding of a TF to target gene promoters would occur prior to the observed change in the expression levels of the TF's target genes. With that in mind, we performed a time-lagged correlation analysis using the optimal time-shift for each motif/cluster combination. With few exceptions, the highest scoring motifs for each cluster show very good correlation of Clover raw score and cluster expression (S2 Table).

**Fig 5:**
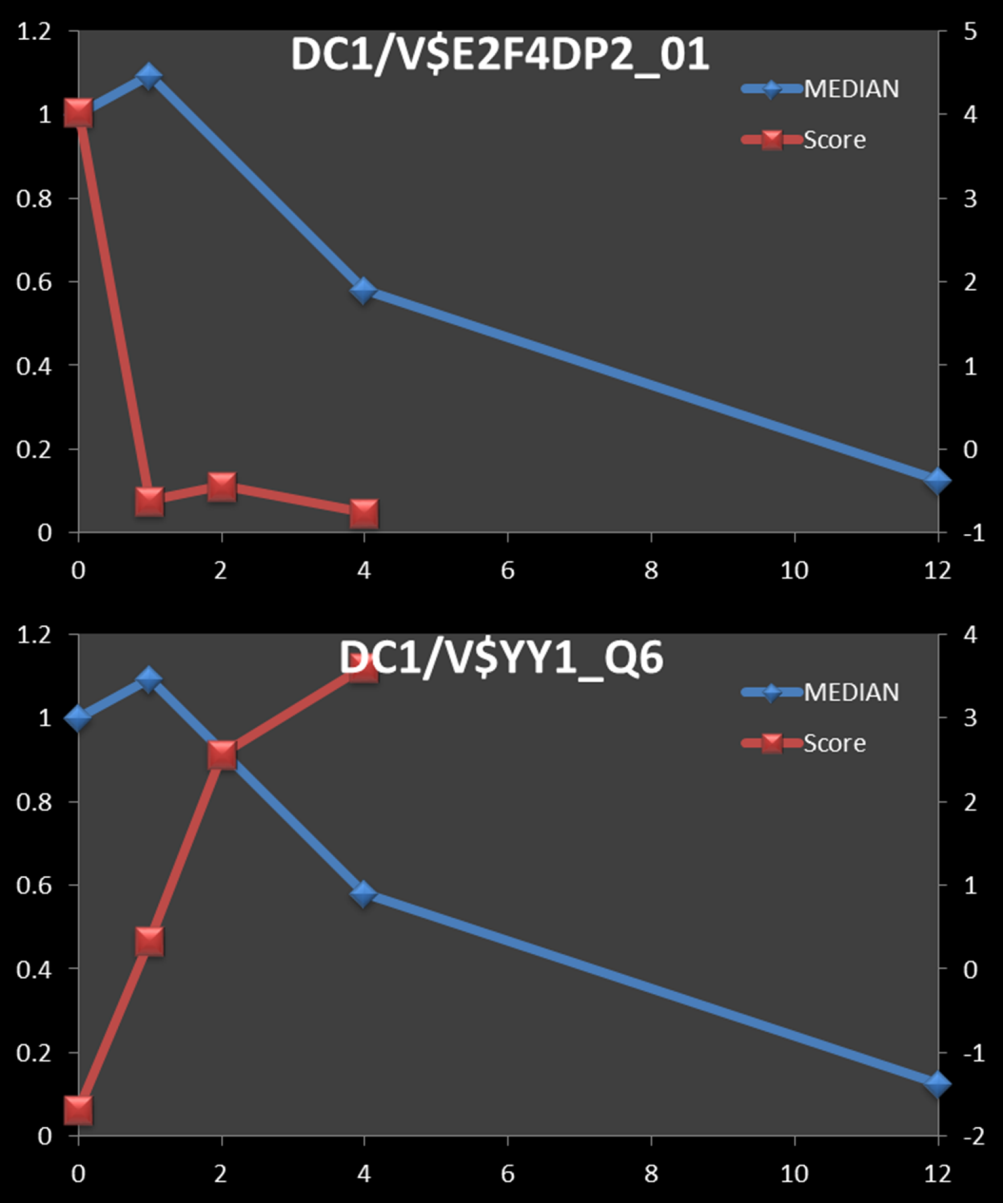
Negative correlation of motif Clover scores and median cluster expression. Blue line and Y axis on the left show median fold change of 122 transcripts in down-regulated cluster 1 (DC1). Clover raw scores for the motifs V$E3F4DP2_01 (*R* = 0.821 at 2 min time shift) (A) and V$YY1_Q6_02 (*R* = -0.999) (B) are represented by red lines and the Y axes on the right.

Next, we assessed if our TF-cluster associations are consistent with current knowledge of TFs that are involved in the macrophage response to LPS-stimulation. For a number of well-characterized TFs, the Clover score and cluster-median expression data showed time-lagged correlations that are consistent with known roles of these factors. For instance, based on TF-gene interactions reported in the Ingenuity Pathways Analysis (IPA) database, the top three TFs associated with the 122 genes in down-regulated cluster 1 (DC1) are E2F4, TP53 and YY1. Consistent with this, the Clover scores for the E2F4 motif showed a strong positive correlation (R = 0.8206 with a +2 hour time shift) with the median DC1 cluster expression (Fig 5A), while those of YY1, a known transcriptional repressor, were strongly anti-correlated (R = -0.9985 with a +2 hour shift) with the median DC1 cluster expression (Fig 5B). We should note that our analysis did not show any of the TP53 motifs as over-represented at any time points for DC1 cluster. Further, by IPA analysis, the DC1 cluster is enriched for genes that are involved in cell cycle and DNA repair pathways, consistent with the known functions of E2F4 (cell cycle; [45]) and YY1 (DNA damage response; [46]). Finally, NFY, the TF whose binding site sequence matches are most highly correlated with the median expression of DC1 (V$NFY_01) (Fig 4), is known to regulate the cell cycle [47].

### Validating TF-cluster temporal associations from our model, using temporal TF occupancy data

To further validate this approach, we tested our predicted associations for three transcription factors (IRF1, IRF8 and SPI1) with the clusters described above (Fig 1) using temporally resolved genome-wide location data from LPS-stimulated macrophages obtained from GEO (GSE56123 [48]). These data consist of ChIP-seq measurements of bone-marrow-derived macrophages at 0, 2, and 4 hours following stimulation with LPS using antibodies against each of these TFs.

**Fig 6:**
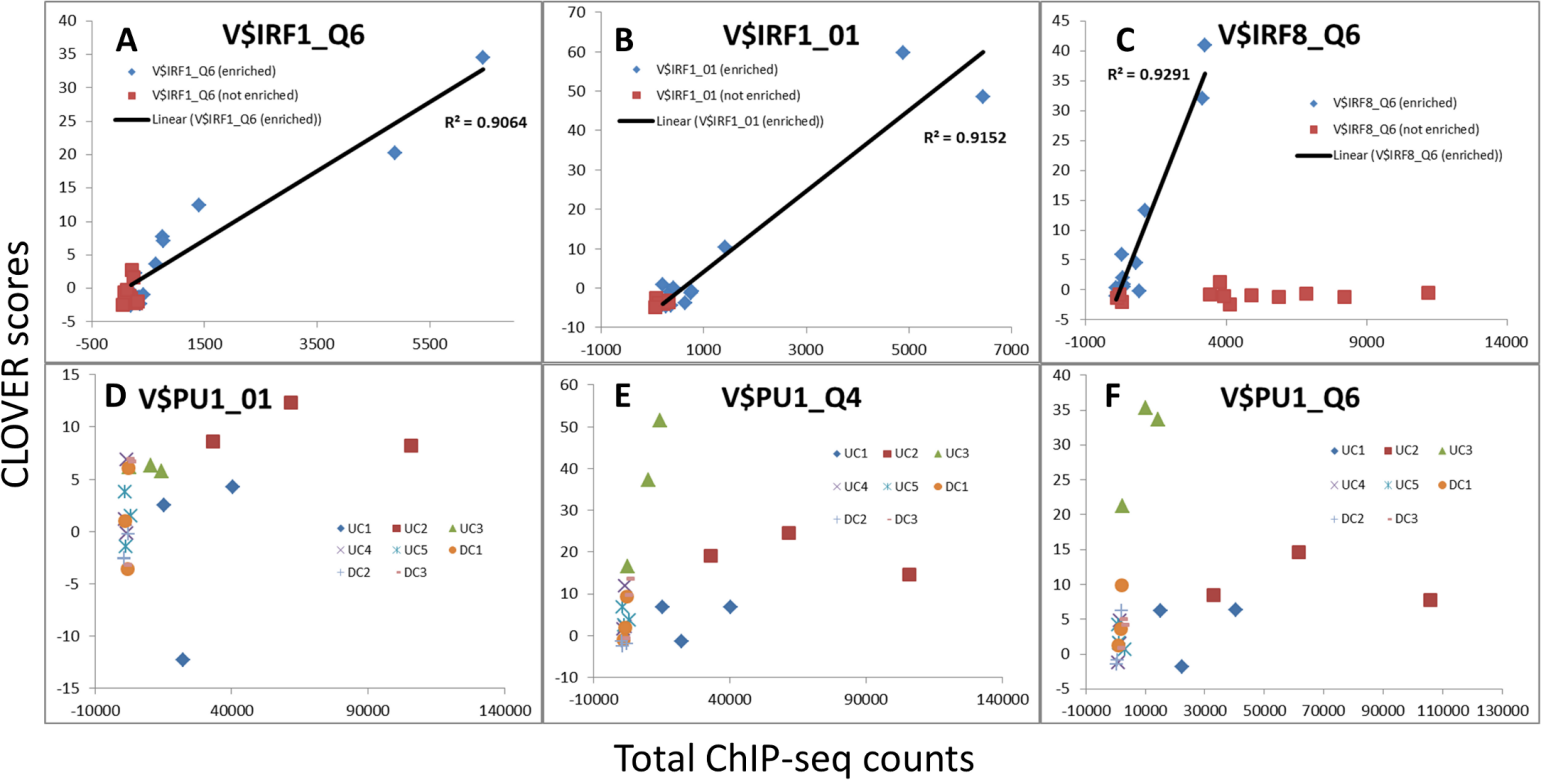
Correlation between Clover scores and observed TF binding. Plots show relationship between Clover scores (y axes) and ChIP-seq counts (x axes) for motifs for IRF1 (V$IRF1), IRF8 (V$IRF8) and PU.1/SPI1 (V$PU1) for all eight clusters at 0, 2 and 4 h time points. Panes A-C show Clover scores and observed counts for enriched motifs (blue diamonds), correlation for those (black line) and Clover scores and counts for motifs that are not enriched (red squares). Panes D-F show Clover scores and ChIP-seq counts for the SPI1 motif separately for each cluster (three time points for each cluster).

To test whether the Clover motif score for each gene cluster correlates with the observed binding of the corresponding TF to gene promoters in the cluster, we compared the motif scores in HAc valleys of genes in 8 clusters against the ChIP-seq signal in the same regions at the identical time points (0, 2 and 4 hours). For all three TFs tested (IRF1, IRF8, and SPI1) the Clover scores were strongly correlated with the appearance of ChIP-seq TF binding signals in the promoters at the time-points predicted to be enriched for the corresponding motif (based on our combined transcriptome and HAc valley analysis approach) (Fig 6). For most clusters in which these motifs were not enriched, both the scores and the observed counts change only negligibly (Fig 6). An exception was noted in case of IRF8, where observed ChIP-seq tag counts in not-enriched clusters were found to increase without a corresponding increase in Clover score (Fig 6); we hypothesize that these data reflect IRF8 binding to a motif that is not included in the TRANSFAC database that we used.

**Fig 7:**
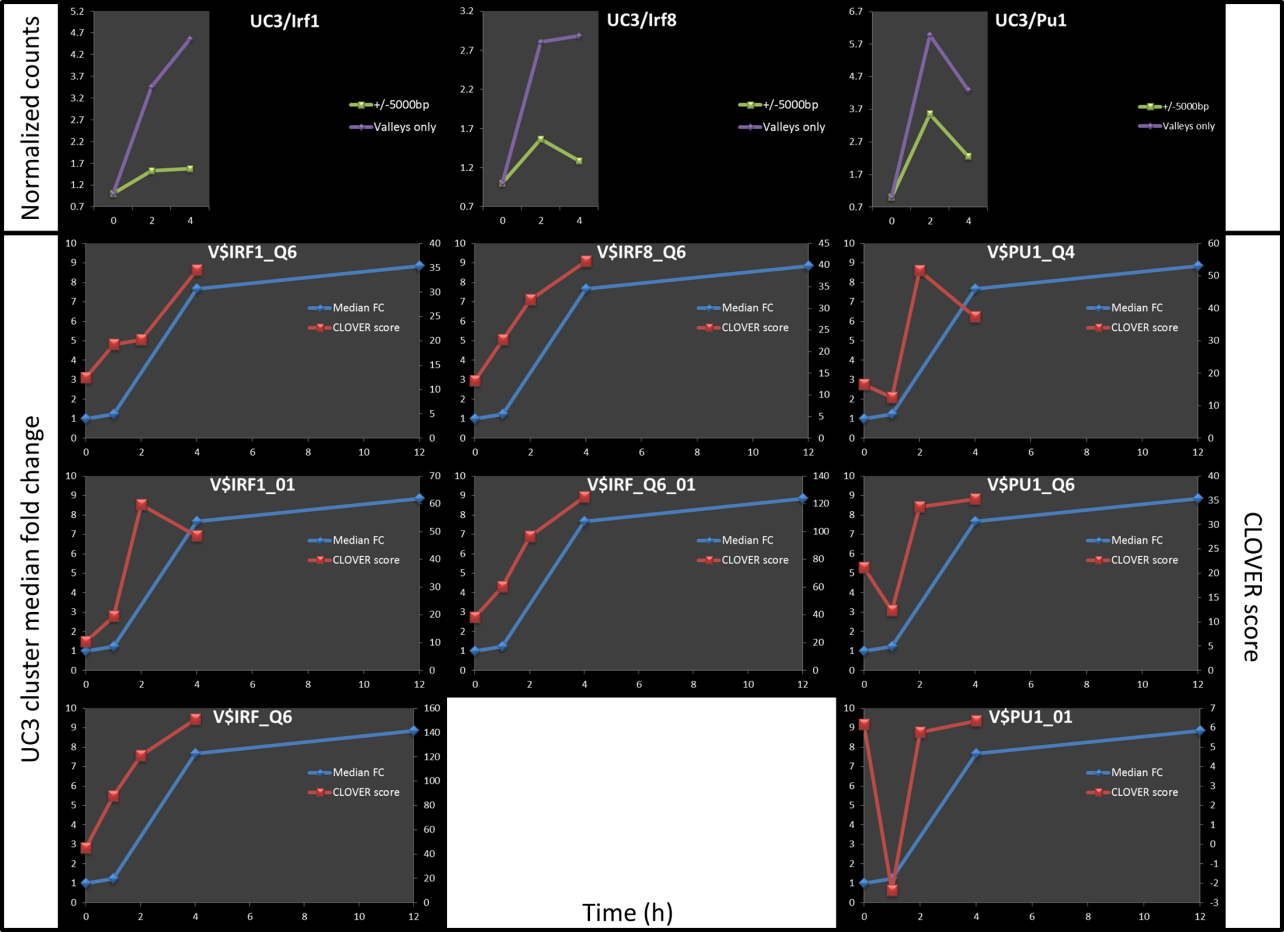
An example of good correlation between predicted and measured TF binding (for the cluster UC3). Top 3 graphs show normalized counts for IRF1, IRF8 and SPI1 within the HAc-valley regulatory elements of genes in DC3 (purple line), or within 10kb region centered at TSS for the same genes (green line). Graphs below show predicted binding of those TFs as represented by Clover raw scores (red lines) superimposed on the UC3 cluster median fold change (blue lines).

**Fig 8:**
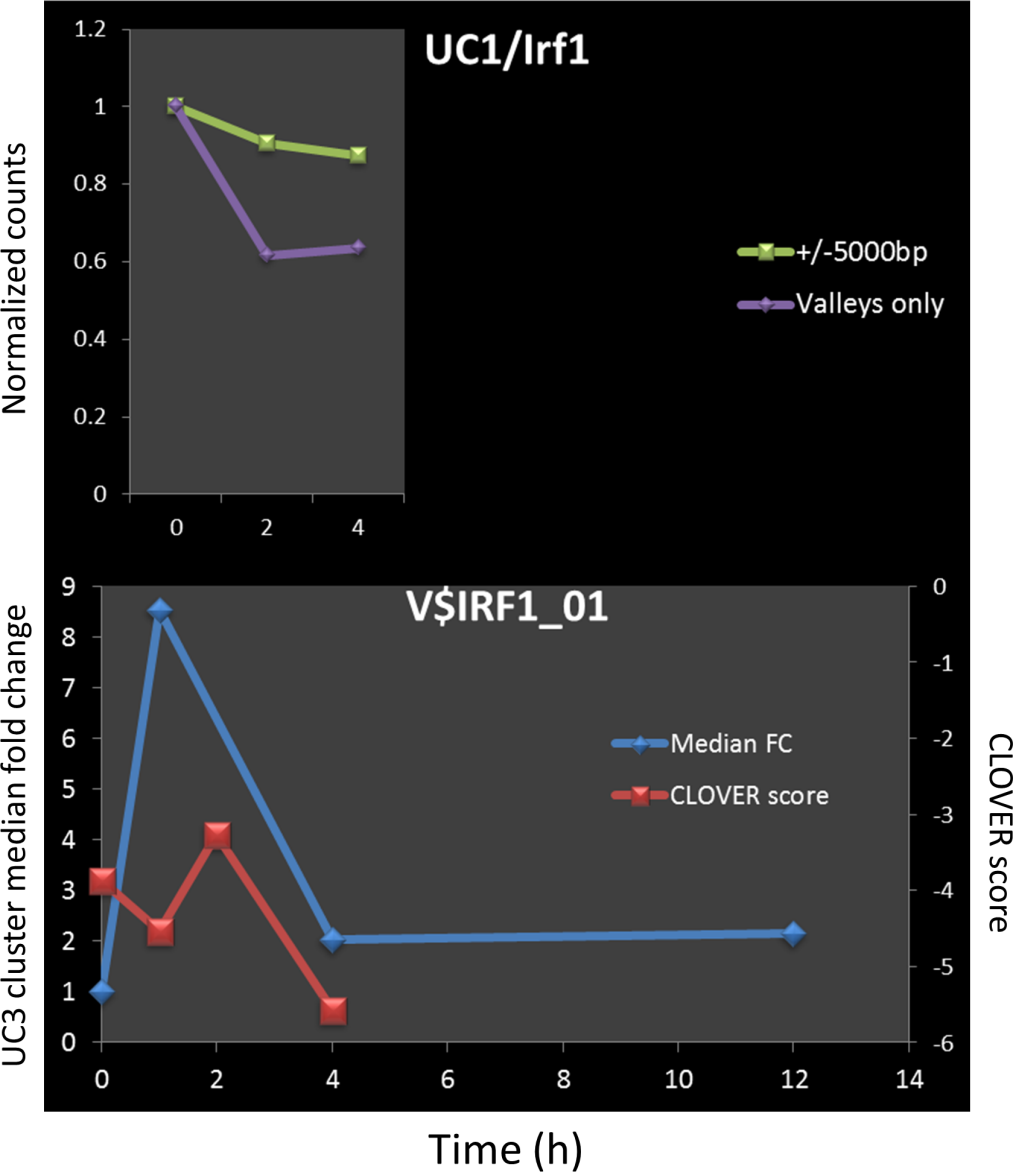
Clover scores and ChIP-seq counts for TF when motif is not over represented. An example of observed counts (pane A) and predicted scores (pane B) for TF whose motif was not found to be over represented. Top graph shows normalized counts for IRF1 within the HAc-valley regulatory elements of genes in UC1 (purple line), or within 10kb region centered at TSS for the same genes (green line). Graph below shows predicted binding of those IRF1 as represented by Clover raw scores (red line) superimposed on the UC1 cluster median fold change (blue line).

In general, the Clover scores and measured binding of the corresponding TFs correlate coherently over time for gene clusters in which the motif is enriched (e.g. IRF1, IRF8, and SPI1 for UC3 –Fig 7) and appear unrelated for clusters in which the motif is not predicted to play a regulatory role (e.g. IRF1 / UC1) (Fig 8). In contrast, IRF1 counts in promoters of genes in UC1 (the cluster for which the IRF1 motif is not enriched) show marginal change both by observation (Fig 8A) and by prediction (Fig 8B).

**Fig 9:**
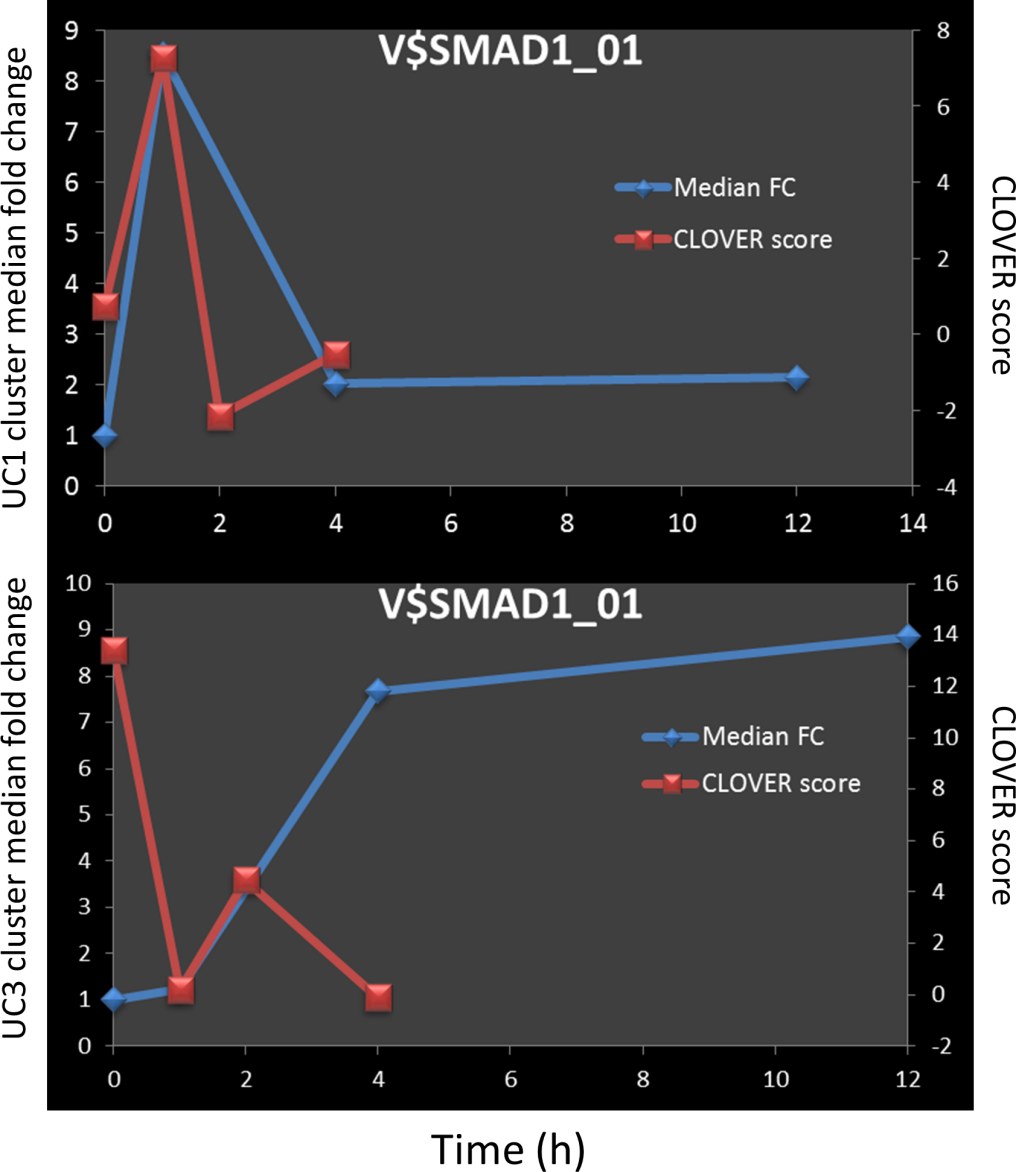
Clover scores and ChIP-seq counts for SMAD1. Median fold change for clusters is shown by blue lines for UC1 (upper pane) and UC3 (lower pane) and the values are shown on the left Y axes. Red lines represent Clover raw scores for V$SMAD1_01 motif and the values are shown on the Y axes on the right.

While a number of TFs with a known role in inflammation were identified using correlation method (e.g. IRF1, IRF8 and SPI1 –Fig 7), we have also flagged novel ones as well. The SMAD family member (SMAD1 –Fig 9) was shown to have a high degree of correlation with the gene expression (0.70 and -0.76 for UC1 and UC3, respectively).

To further the TF-gene cluster associations derived using the HAc valley data, we examined the ChIP-seq signal in the unmasked promoter regions (-2000 to +500 bp relative to the TSS) of genes in each cluster (Fig 1). We compared the total number of ChIP-seq reads for each TF in the promoter regions of the genes in each of the eight clusters with the distributions of summed-counts for randomly selected sets of expressed genes identical in size to each cluster. We tested the summed-counts for the gene cluster for extremality in the distribution of summed-counts based on the randomly constructed gene sets, yielding an enrichment *p*-value for each combination of ChIP-seq experiment and gene cluster. For clusters where a TF was predicted to be enriched, we observed significantly higher counts as compared to random distribution (Table 1). For instance, IRF8 binding at 2 hours was strongly enriched with the DC1 genes (*q* < 0.01); DC3 genes (*q* < 0.01) (Table 1).

**Table 1:** Enrichment test results for ChIP-seq tags for specific TFs in HAc-valley regulatory elements within ±5 kbp of TSSs for genes in specific clusters.

TF	ChIP-seq Time point	Cluster	*predicted*	*q-*value
IRF8	0 h	DC1	yes	5.6×10^−3^
IRF8	0 h	DC3	no	9.5×10^−3^
IRF8	2 h	UC2	yes	2.3×10^−7^
IRF8	4 h	UC2	yes	6.4×10^−9^
IRF8	0 h	UC3	yes	1.4×10^−11^
IRF8	2 h	UC3	yes	5.2×10^−25^
IRF8	4 h	UC3	yes	2.0×10^−30^
IRF1	2 h	UC2	yes	4.6×10^−3^
IRF1	4 h	UC2	yes	9.8×10^−3^
IRF1	0 h	UC3	yes	3.7×10^−28^
IRF1	2 h	UC3	yes	1.4×10^−24^
IRF1	4 h	UC3	yes	2.9×10^−25^
IRF1	2 h	UC5	yes	3.7×10^−2^
IRF1	4 h	UC5	no	4.6×10^−2^
SPI1	0 h	DC3	yes	4.9×10^−3^
SPI1	0 h	UC2	yes	7.1×10^−4^
SPI1	2 h	UC2	yes	9.6×10^−12^
SPI1	4 h	UC2	yes	6.2×10^−10^
SPI1	2 h	UC3	yes	4.8×10^−8^
SPI1	4 h	UC3	yes	2.7×10^−10^

The first two columns (“TF” and “ChIP-seq time point”) identify the specific ChIP-seq dataset; the “Cluster” column identifies the gene expression cluster with which the TF was predicted to be associated; the “predicted” column indicates whether or not the TF’s binding site motif was statistically enriched (*p* < 0.01) in HAc-valley regulatory elements based on H4ac ChIP-seq data at the indicated time point; and the “*q*-value” column gives the q-value for the enrichment test for the TF-specific ChIP-seq experiment for the indicated cluster at the indicated time point.

## Discussion

Regulation of gene expression in mammals is combinatorial, tissue/context-specific, and dynamic. A substantial portion of the regulation of gene activity is performed at the level of transcription. With the increase in the evolutionary complexity of species, the number of genes that the species’ genome encodes for, does not seem to correlate with the evolutionary complexity. Estimates for the number of human protein-coding genes have been continuously lowered and the best current guess is around 20,000, a far cry from some of the earlier estimates of more than 100,000 [49]. The number of genes in humans is higher than in other mammals but lower than that in many plants [50]. Converging lines of evidence suggest that the complexity of higher organisms arises in part from the intricate, multifactorial, gene regulation and complex gene product interactions, rather than from the sheer number of available genes [51].

Methods employing knockout (KO) mice, transient or permanent KO, knockdown, or overexpression of a gene *in vitro*, allow for relatively easy detection of genes involved in a certain phenotype in situations where the pathway regulating the phenotype has a single gene bottleneck. However, almost no phenotype in higher organisms is a product of one gene, even in the situation where a bottleneck exists. While *Tlr4*^-/-^ mice do not respond to LPS, response to LPS is dependent on thousands of genes that are carefully regulated and function in concert to produce a complex response ([52–54]). Those genes (regulators and effectors) would be expected to be under tight evolutionary pressure meaning that they work together to provide an appropriate response to pathogens. A deficiency in LPS response in macrophages would be expected to impair the innate immune response to pathogenic challenge, while over-response could lead to tissue damage and autoimmune dysfunction. It is reasonable to assume that the gene regulatory network downstream of TLR4 in macrophages represents an evolutionary compromise in which changing a single, or even a few genes, results only in a modest modulation of response. While those differences are almost certainly evolutionary and functionally important, experimentally demonstrating their functional significance can be challenging.

The method described in this manuscript offers one approach to exploring a complex interplay of multiple transcription factors that are involved the regulation of gene expression. While in itself, this method does not prove involvement of any particular transcription factor, it computationally predicts candidate TFs of which our results indicate a significant proportion undergo LPS-dependent changes in TF binding to target gene clusters. Even after stringent *p* value filtering, a number of motifs still remain significant for each cluster. While many of those motifs are associated with transcription factors already known to be important during the inflammatory response in macrophages, quite a few appear to be novel (S1 Table). Several different methods were used to sort motifs in order to allow the most significant ones to rise to the top. Three principles guided our efforts: 1. Higher Clover raw score correlates with significant binding; 2. Motifs that show large Clover raw scores differences between time points are active in regulating genes for that cluster and 3. Correlation (positive or negative) between Clover raw score and cluster median expression suggests a direct role of those motifs in regulating that particular set of genes. To account for 1 and 2, we have sorted the hits either by highest score at any time point, highest score at the time point closest to the highest cluster expression or by highest total score difference (d = MaxScore-MinScore). All of these three methods produced similarly sorted lists. In contrast, sorting by correlation, especially if time shift was introduced, produced significantly different result (S2 Table). Considering that the epigenetic changes and TF binding and dissociation can occur rather quickly, on the scale of minutes, the concordance between the list of TFs sorted by time-lagged correlation and the other three TF sorting heuristics would be expected to improve with higher-resolution temporal transcriptome profiling.

Most of the high ranking transcription factors predicted to play a role in LPS response, by the method presented here have previously been described as having a role in inflammation. In addition to those however, a number of additional transcription factors, not previously described in the context of inflammation, were found to have a high degree of correlation with the gene expression. Sequence matches to the SMAD1 binding motif were found to be enriched in the active promoter regions of clusters UC1 and UC3 (Fig 9). At first glance, it would appear that this TF is acting as an activator for the genes in UC1, and as a repressor for the genes in UC3. However, the temporal resolution of the transcriptome profiling is not sufficient to draw that conclusion in the case of UC1. In the case of UC3, since the cluster-median gene expression increases and reaches a plateau, and since the SMAD1 Clover score goes down and stays down, it is reasonable to suppose that it acts as a repressor.

The transcription factor SMAD1 is activated by bone morphogenic protein type 1 (BMP1) receptor kinase ([55, 56]). We found that in our data, *Bmp1* transcript was transiently upregulated after LPS stimulation (S1 Fig). Recently, it has been reported that SMAD1/5 pathway can be activated by TGF-B1 in human primary macrophages, and is not affected by bone morphogenic proteins [57]. TGF-B1 is known to inhibit the inflammatory response of macrophages to LPS, an effect which was found to be specifically mediated through SMAD3 [58]. While in general stimulation of macrophages by TGF-B1 is anti-inflammatory [58], SMAD1/5 activation by TGF-B1 promotes pro-inflammatory, pro-atherogenic effect [57].

## Supporting information

S1 Fig Expression of Bmp1 transcript in LPS stimulated macrophages. Bars show normalized expression levels of Bmp1 transcript in unstimulated macrophages and at 1, 4 and 12h post LPS stimulation.
(TIF)

S1 Table Results for motif scanning in APRs of eight expression clusters for all time points. Each tab shows all motifs for one expression cluster that were found to be significantly over-represented at one time point at least. Columns labeled as “Score” represent Clover score and “*p* value” represent over-representation *p* value (relative to the background set of genes). MaxScoreDifference column represents Clover score difference between highest and lowest value (at any time point).
(XLSX)

S2 Table Correlation of motif scores and cluster fold change. Clover raw score are shown for time points 0, 1, 2 and 4h for each motif and each cluster (7,272 total). Cluster median expression is shown for each cluster. Three different correlation scores are shown, one without any time lag, one for fixed time lag (fixed for each cluster) and one for optimal time lag (best score) for that motif/cluster combination.
(XLSX)
