# Supplementary figures and images for: Identifying novel transcription factors involved in the inflammatory response by using binding site motif scanning in genomic regions defined by histone acetylation
Source: PLoS One. 2017 Sep 18;12(9):e0184850. doi: 10.1371/journal.pone.0184850 (PMC5602638; doi:10.1371/journal.pone.0184850)

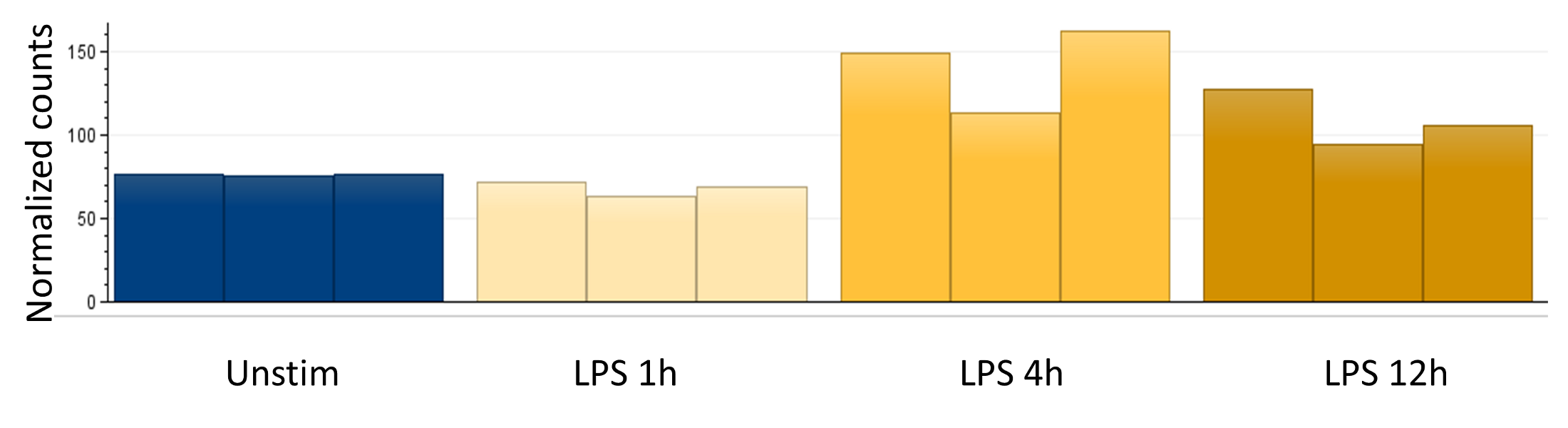

Supplement: S1 Fig — Bars show normalized expression levels of Bmp1 transcript in unstimulated macrophages and at 1, 4 and 12h post LPS stimulation. (TIF) [file pone.0184850.s001.tif]
